# Supplementary material for: Mapping genomic and transcriptomic alterations spatially in epithelial cells adjacent to human breast carcinoma
Source: Nat Commun. 2017 Nov 1;8:1245. doi: 10.1038/s41467-017-01357-y (PMC5665998; doi:10.1038/s41467-017-01357-y)
Supplement: Supplementary file 1 — Supplementary Information [file 41467_2017_1357_MOESM1_ESM.pdf]

Supplementary Figure 1.

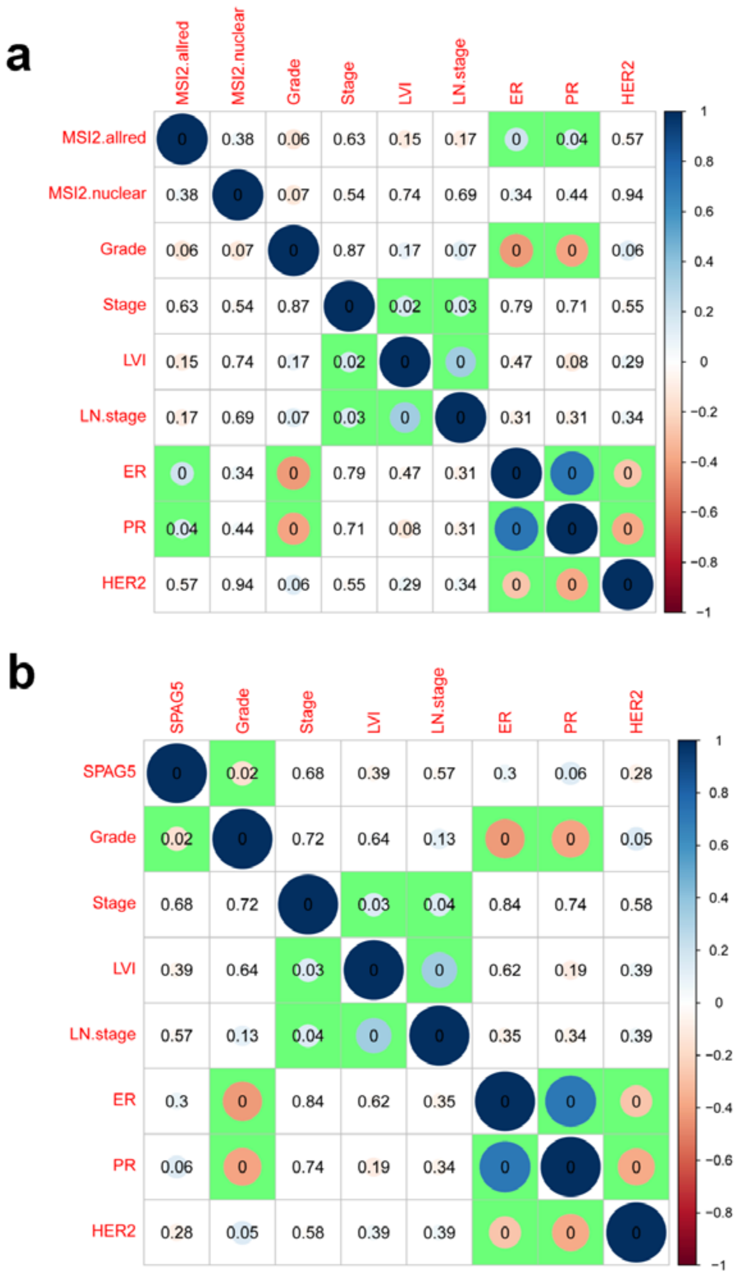

Supplementary Figure 1. Correlation matrices for **(a)** MSI2 and **(b)** SPAG5 TMAs. LVI denotes Lymphovascular invasion; LN.stage indicates lymph node stage. A green background denotes significant correlations for MSI2 and SPAG5. The legend on the side highlights the direction/magnitude of the significant correlations; the number in each box denotes the p-value for the corresponding correlation.

Supplementary Figure 2.

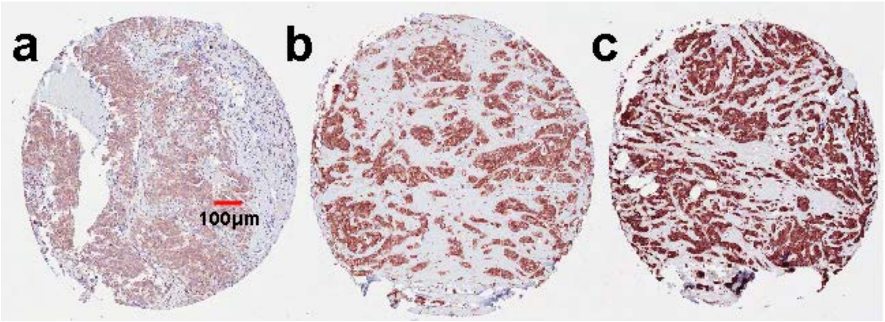

Supplementary Figure 2. MSI2 TMA Allred categorization **(a)** Allred score 6. **(b)** Score 7. **(c)** Score 8.

Supplementary Figure 3.

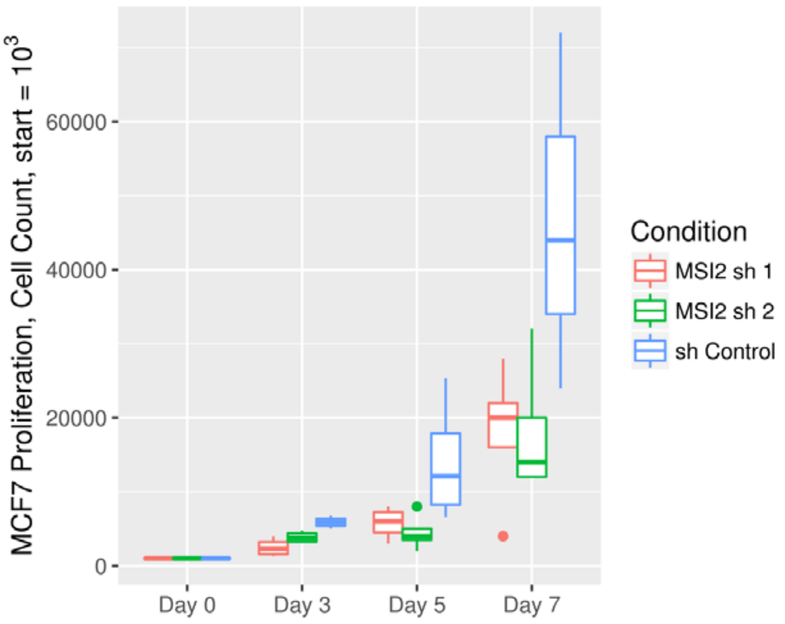

Supplementary Figure 3. ***MSI2*** **overexpression increases proliferative capacity of MCF7** MCF7 shRNA control and shRNA *MSI2* clones counted by Vi-cell-XR and equally plated to assess proliferation by trypan blue. *MSI2*-GFP clones demonstrated a significant decrease in proliferative capacity 5 and 7 days following transfection (Welch's t-test; p-value < 0.05).

SUPPLEMENTARY TABLES

Supplementary Table 1. Tabulated summary of variance (adjusted r^2; to 4 significant digits) explained by copy number aberrations, after adjusting for baseline expression (i.e. O1, the contralateral duct epithelial sample). This analysis was not possible for patient 4 (where samples O1 and D1 were outliers and excluded) and patient 10 (where sample D1 and D2 were also excluded as outliers). Across all patients, copy number aberrations explain little of the perturbation observed in gene expression data associated with increasing proximity to tumour. Notably, however, copy number aberrations can explain a small part of the expression perturbations observed in tumours.

Patient	D1	D2	T
2	0	0	0.0007
4	---	---	---
6	0.0047	0.0004	0.0041
8a	0.0059	0.0008	0.0034
8b	0	0.0002	NA
9	0	0	0.01
10	---	---	---
14	0.0061	0	0.0025
22	---	0.0015	0.024

Supplementary Table 2. For two groups of genes (genes that positively correlate with proximity to tumour and all other bicluster genes), we can separate them according to the absence or presence of at least one somatic mutation in at least one breast cancer tumour from 8 different breast cancer cohorts, including The Cancer Genome Atlas (TCGA)[1,2], METABRIC[3], and datasets from the Broad[4], Sanger[5], and British Columbia Cancer Research Centre[6] (curated by the cBIO Cancer Genomics Portal[7]). *We limited our analysis to genes that were included in the bicluster analysis (i.e. top 30% of univariately informative genes; see* ***Text****) and assessed in at least one cohort.* We noted that genes in modules that correlate (positively and) significantly with proximity to tumour were more likely to be mutated in breast cancer patients across all eight datasets (Chi-square test; Yates p-value < 0.05; odds ratio = 1.33; 95% confidence interval = (1.178, 1.5115)).

	**Genes with no Somatic Mutations**	**Genes with at least 1 Somatic Mutation**
Gene sets that positively correlate with proximity to tumour	467	1517
All other bicluster genes	1135	2763

Supplementary Table 3. For two groups of genes (genes that negatively correlate with proximity to tumour and all other bicluster genes), we can separate them according to the absence or presence of at least one somatic mutation in at least one breast cancer tumour from 8 different breast cancer cohorts, including The Cancer Genome Atlas (TCGA)[1,2], METABRIC[3], and datasets from the Broad[4], Sanger[5], and British Columbia Cancer Research Centre[6] (curated by the cBIO Cancer Genomics Portal[7]). *We limited our analysis to genes that were included in the bicluster analysis (i.e. top 30% of univariately informative genes; see **Text**) and assessed in at least one cohort.* As in Supplementary Table 4, modules that negatively and significantly correlate with proximity to tumour were enriched for genes mutated in breast cancer (Chi-square test; Yates p-value < 0.05; odds ratio = 1.18; 95% confidence interval = (1.0146, 1.383)).

	**Genes with no Somatic Mutations**	**Genes with at least 1 Somatic Mutation**
Gene sets that negatively correlate with proximity to tumour	254	781
All other bicluster genes	1348	3499

SUPPLEMENTARY REFERENCES

1 Ciriello, G. *et al.* Comprehensive molecular portraits of invasive lobular breast cancer. *Cell* **163**, 506-519 (2015).

2 Network, C. G. A. Comprehensive molecular portraits of human breast tumors. *Nature* **490**, 61 (2012).

3 Pereira, B. *et al.* The somatic mutation profiles of 2,433 breast cancers refines their genomic and transcriptomic landscapes. *Nature communications* **7** (2016).

4 Banerji, S. *et al.* Sequence analysis of mutations and translocations across breast cancer subtypes. *Nature* **486**, 405-409 (2012).

5 Stephens, P. J. *et al.* The landscape of cancer genes and mutational processes in breast cancer. *Nature* **486**, 400-404 (2012).

6 Shah, S. P. *et al.* The clonal and mutational evolution spectrum of primary triple-negative breast cancers. *Nature* **486**, 395-399 (2012).

7 Cerami, E. *et al.* (AACR, 2012).
