# Supplementary material for: Mapping genomic and transcriptomic alterations spatially in epithelial cells adjacent to human breast carcinoma
Source: Nat Commun. 2017 Nov 1;8:1245. doi: 10.1038/s41467-017-01357-y (PMC5665998; doi:10.1038/s41467-017-01357-y)
Supplement: Supplementary file 3 — Description of Additional Supplementary Files [file 41467_2017_1357_MOESM3_ESM.pdf]

Description of Additional Supplementary Files

File Name: Supplementary Data 1
Description: Tabulated clinico-pathological features for patients included in this study. Patient # refers to the patient study number. Histology denotes the type of invasive breast cancer: IDC = invasive ductal carcinoma; ILC = invasive lobular carcinoma; PILC = pleomorphic invasive lobular carcinoma; and DCIS = ductal carcinoma in situ. Grade was scored using a modified Bloom and Richardson grade of the invasive carcinoma: 2 = intermediate grade; and 3 = high grade. A is the architecture (degree of tubule formation) and was scored as 1, 2 or 3. N denotes the degree of nuclear pleomorphism and is scored as 1, 2 or 3. M is the mitotic count (scored as 1, 2 or 3). Size refers to the size of invasive carcinoma in cm. LVI denotes lymphovascular invasion. Focus is the number of foci of invasive carcinoma. LN is the number of lymph nodes with metastases: itc = isolated tumour cells; mic = micrometastasis of 0.2 - 2.0 mm. T denotes the pT stage and N refers to the pN stage. Stage indicates the extent of disease spread in the patient. Abbreviations: DCIS, ductal carcinoma in situ; ER, estrogen receptor; PR, progesterone receptor.

File Name: Supplementary Data 2
Description: Tabulated relative log ratio of enrichment/depletion of all genes across all samples.

File Name: Supplementary Data 3
Description: Gene-to-module mapping.

File Name: Supplementary Data 4
Description: List of genes in module 16.

File Name: Supplementary Data 5
Description: Pathway enrichment results for module 16.

File Name: Supplementary Data 6
Description: List of genes in module 2.

File Name: Supplementary Data 7
Description: Pathway enrichment results for module 2.
